# Sphingolipids as critical players in retinal physiology and pathology

**DOI:** 10.1194/jlr.TR120000972

**Published:** 2021-02-06

**Authors:** M. Victoria Simon, Sandip K. Basu, Bano Qaladize, Richard Grambergs, Nora P. Rotstein, Nawajes Mandal

**Affiliations:** 1 Instituto de Investigaciones Bioquímicas de Bahía Blanca (INIBIBB), Departamento De Biología, Bioquímica y Farmacia, Universidad Nacional del Sur (UNS), Argentine National Research Council (CONICET), Bahía Blanca, Argentina; 2 Departments of Ophthalmology and Anatomy and Neurobiology, University of Tennessee Health Science Center, Memphis, TN, USA

**Keywords:** ceramide, sphingosine-1-phosphate, ceramide-1-phosphate, photoreceptor degeneration, age-related macular degeneration, retinitis pigmentosa, ADIPOR1, adiponectin receptor 1, AH, aqueous humor, AMD, age-related macular degeneration, ASAH, *N*-acyl-sphingosine amidohydrolase, aSMase, acid SMase, *BEST1*, bestrophin-1, BDNF, brain-derived neurotrophic factor, CDase, ceramidase, Cer, ceramide, CerK, ceramide kinase, CERKL, ceramide kinase-like, CerS, ceramide synthase, CFH, complement factor H, C1P, ceramide 1-phosphate, DHCer, dihydroceramide, DR, diabetic retinopathy, EAU, experimental autoimmune uveoretinitis, GA, geographic atrophy, GalCer, galactosylceramide, GCS, glucosylceramide synthase, GlcCer, glucosylceramide, hBest1, human bestrophin-1, HexCer, hexosylceramide, IOP, intraocular pressure, LacCer, lactosylceramide, Mac Tel, macular telangiectasia, NGF, nerve growth factor, nSMase, neutral sphingomyelinase, OAG, open-angle glaucoma, PARP-1, poly-ADP ribose polymerase 1, PDR, proliferative diabetic retinopathy, PKC, protein kinase C, POAG, primary open-angle glaucoma, RP, retinitis pigmentosa, RPE, retinal pigment epithelium, SMS, SM synthase, Sph, sphingosine, SphK, sphingosine kinase, S1PR, S1P receptor, SPT, serine palmitoyl transferase, VEGF, vascular endothelial growth factor, VMD, vitelliform macular dystrophy

## Abstract

Sphingolipids have emerged as bioactive lipids involved in the regulation of many physiological and pathological processes. In the retina, they have been established to participate in numerous processes, such as neuronal survival and death, proliferation and migration of neuronal and vascular cells, inflammation, and neovascularization. Dysregulation of sphingolipids is therefore crucial in the onset and progression of retinal diseases. This review examines the involvement of sphingolipids in retinal physiology and diseases. Ceramide (Cer) has emerged as a common mediator of inflammation and death of neuronal and retinal pigment epithelium cells in animal models of retinopathies such as glaucoma, age-related macular degeneration (AMD), and retinitis pigmentosa. Sphingosine-1-phosphate (S1P) has opposite roles, preventing photoreceptor and ganglion cell degeneration but also promoting inflammation, fibrosis, and neovascularization in AMD, glaucoma, and pro-fibrotic disorders. Alterations in Cer, S1P, and ceramide 1-phosphate may also contribute to uveitis. Notably, use of inhibitors that either prevent Cer increase or modulate S1P signaling, such as Myriocin, desipramine, and Fingolimod (FTY720), preserves neuronal viability and retinal function. These findings underscore the relevance of alterations in the sphingolipid metabolic network in the etiology of multiple retinopathies and highlight the potential of modulating their metabolism for the design of novel therapeutic approaches.

## Why sphingolipids?

The notion that lipids are part of cellular signaling networks in addition to their canonical roles as energy reserves or structural membrane components was first proposed in the 1950s and is now widely accepted. However, outside the lipid community, their involvement is still overshadowed by their nonlipidic counterparts. Sphingolipids, one of the three main classes of membrane lipids, are among the latest incorporations to the club of recognized bioactive lipids. They owe their name to the initial enigma regarding their functions, which reminded J. L. Thudichum, who first isolated them from brain tissue during the late 19th century, of the riddle posed by the Sphinx in Greek mythology. After almost a century, this riddle was thought to be resolved when they were classified only as stable membrane structural components. Groundbreaking findings in the mid-1980s and early 1990s revealed novel roles for sphingosine (Sph) and ceramide (Cer) as signaling molecules involved in the induction of cell death and the inhibition of proliferation (1, 2, 3, 4). Later work established that their phosphorylated derivatives, S1P and ceramide 1-phosphate (C1P), promote survival, proliferation, and differentiation (5, 6, 7, 8, 9), thus increasing the repertoire of bioactive sphingolipids. Accumulated evidence has not only expanded this family of bioactive sphingolipids to include glucosylceramide (GlcCer), lactosylceramide (LacCer), and some gangliosides, such as GM1, but has also established sphingolipids as amazingly versatile signaling molecules regulating multiple physiological and pathological processes.

In the last two decades, work from several groups had uncovered key roles for sphingolipids as signaling molecules in the retina. Sphingolipids are now known to modulate the functionality of the multiple cell types present in the retina and, through dysregulation of their metabolism and populations, contribute to multiple retinal pathologies (10, 11, 12). In this review, we first introduce the roles and metabolism of simple sphingolipids, with particular focus on Cer and S1P, and then integrate the roles played by these lipids in several retinal pathologies.

## The complexity of sphingolipid structure and metabolic pathways

**Fig. 1:**
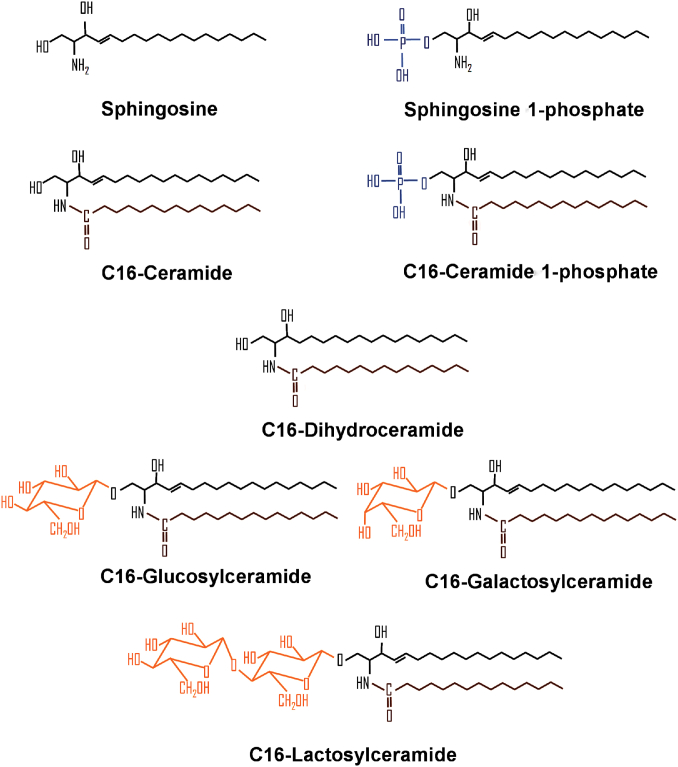
Chemical structures of sphingolipids. The Sph backbone (black) is shared by all sphingolipids. Sph is amide-linked to a fatty acid moiety (brown), forming Cer. Later additions of a phosphate (blue) or hexose residues (orange) give rise to several sphingolipid molecules.

The amazing diversity of sphingolipid structures is the basis for their extraordinary functional versatility. Structurally, sphingolipids are amphipathic molecules that share a hydrophobic region, a sphingoid long-chain (18–20 carbon) base, that constitutes the building block of mammalian sphingolipids. Addition of a fatty acid through an amide bond to carbon 2 of the Sph backbone gives rise to Cer (Fig. 1). Modifications of the sphingoid backbone can generate a large variety of structures. On the other hand, sphingolipid hydrophilic regions can vary widely; the addition of a single phosphate to Sph and Cer generates S1P and C1P, respectively, while attachment of diverse headgroups at the C-1 position of Cer gives rise to more complex sphingolipids, such as SM and glycosphingolipids. The variability and potential combination of these moieties give rise to an astonishing assortment of sphingolipid molecular species. Over 60 sphingoid bases have been reported, varying in their chain length (usually 18–20 carbons), the number of double bonds (often zero to one, but up to two), and number of hydroxyl groups (two to four) (13), with Sph being the most common. Similarly, over 20 fatty acids are found in Cers, differing in their chain lengths (generally 14–36 carbon atoms), unsaturation (typically saturated but occasionally highly unsaturated), and hydroxylation. Finally, the existence of hundreds of headgroups that can be attached to Cer has established the amount of sphingolipid molecular species in the order of tens of thousands (14).

To fully unravel the sphingolipid puzzle, it is necessary to consider their bewildering diversity in the context of their myriad metabolic pathways. The complexity and high interconnection of these pathways are the basis for the crucial roles in controlling numerous cellular functions. The biosynthesis and catabolism of sphingolipids involves multiple metabolic intermediates, many of which have biological functions of their own. The constant flux of cellular sphingolipid levels is the key to their ability to modulate multiple cellular processes. Sphingolipid concentrations vary among the different cell types, with SM being the most abundant. In most cells, their relative ratios can be illustrated as SM (30,000):Cer (3,000):Sph (100):S1P (1). Thus, minor changes in SM levels translate into significant variations in Cer and S1P concentrations, which result in a specific cellular response (15).

**Fig. 2:**
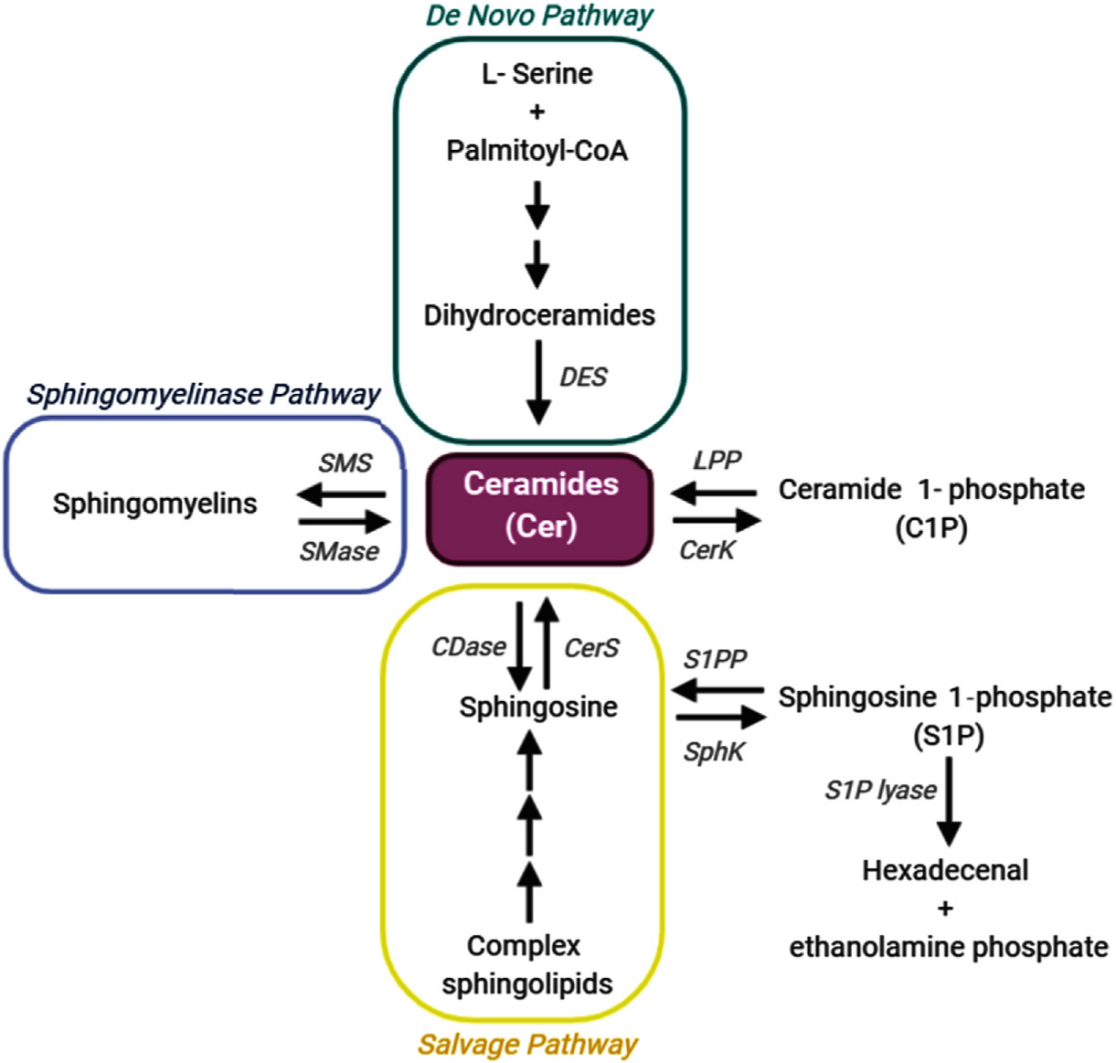
The sphingolipid networks. A schematic view of the interconnected sphingolipid network, which has Cers (purple) forming its central hub. Cer can be synthesized through the de novo pathway (green), initiated by the condensation of l-serine and palmitoyl-CoA; through the SMase pathway (blue), from the degradation of SM catalyzed by different SMases; or through the Salvage pathway (yellow), from the Sph generated by the degradation of complex sphingolipids. Cer can then serve as a substrate for sphingomyelin synthesis by SMS; be phosphorylated by a CerK to generate C1P; or be deacylated by CDases to form Sph, which can in turn be phosphorylated by SphK to produce S1P. S1P can be dephosphorylated by S1P phosphatase (S1PP) to regenerate Sph or be irreversibly degraded by S1P lyase to render ethanolamine 1-phosphate and hexadecenal, an irreversible reaction that provides the only escape pathway from this intricate metabolic network. *DES*, dihydroceramide desaturase-1; LPP, lipid phosphate phosphatases.

Cer is the undisputed hub of the highly interconnected sphingolipid metabolic network and controls key cellular responses such as growth arrest, senescence, and cell death (15). Three different pathways lead to Cer formation: de novo synthesis, hydrolysis of SM, and recycling of Sph and complex sphingolipids (Fig. 2). It is noteworthy that these three pathways are activated by different cellular cues and contribute differentially to Cer signaling capacity. The de novo pathway of Cer synthesis takes place in the ER and starts with the condensation of l-serine and palmitoyl-CoA, catalyzed by l-serine palmitoyl transferase (SPT), to form 3-ketosphinganine, which is then reduced to sphinganine (Fig. 2). Next, Cer synthases (CerSs) catalyze the N-acylation of sphinganine, giving rise to dihydroceramides (DHCers). In mammals, the CerS family is formed by six isoforms (CerS1–6), which differentially utilize fatty acylCoAs differing in chain length from 14 to 34 carbons. DHCer desaturases then reduce DHCer to yield a diversity of Cer species (16). The de novo pathway is activated upon different environmental events to induce stress responses and cell death (17, 18). Once generated, Cer can then be transferred to the Golgi, either through a Cer transporter (CERT) or through vesicular transport pathways, where it serves as a precursor for SM, C1P, or other glycosphingolipids. SM is synthesized through the addition of a phosphorylcholine to Cer, catalyzed by SM synthase (SMS) (19); the resulting SM is then conveyed to the plasma membrane through vesicular transport. In turn, the phosphorylation of Cer, catalyzed by Cer kinase (CerK), produces C1P (20, 21).

Glycosphingolipids are formed from Cer by sequential addition of sugar moieties; the addition of glucose or galactose to Cer gives rise to GlcCer and galactosylceramide (GalCer), respectively, which are two structural isomers collectively called hexosylceramides (HexCers) (22). Their biosynthetic mechanism is similar: a glucosyltransferase (GlcCer synthase) transfers UDP-glucose to Cer, forming GlcCer, while a galactosyltransferase transfers UDP-galactose to Cer, generating GalCer (23). They both serve as precursors for more complex sphingolipids, such as LacCer, which is the precursor for most gangliosides. The synthesis of these sialic acid-containing glycosphingolipids starts with the attachment of a sialic acid to LacCer, generating GM3, which serves as an essential core structure for the building of the complex oligosaccharide chains found in gangliosides. These sequential glycosylation reactions, catalyzed by different glycosyltransferases, take place mainly in the luminal surface of the Golgi and in *trans*-Golgi membranes (24, 25, 26).

Complex sphingolipids are distributed to the plasma membrane and different subcellular compartments and can, in turn, be catabolized to serve as sources of Cer in basal or signal-activated intracellular pathways. Earlier work identified signaling roles of Cer generated by a group of SM-hydrolyzing enzymes called SMases, which produce Cer via hydrolysis of the SM phosphodiester bond and consequent release of the phosphorylcholine headgroup. This mechanism of Cer generation is called the SMase pathway (Fig. 2). There are at least five different isoforms of SMase that differ in their cationic dependence, pH optimum, and subcellular localization, and are found in the plasma membrane, cytosol, mitochondria, and endo-lysosomal compartments (27, 28, 29). Acid SMase (aSMase) catalyzes the hydrolysis of SM present in the endo-lysosomal compartments and the outer leaflet of the plasma membrane, as well as SM carried by lipoproteins (30). At least four neutral SMases (nSMases) have been identified, with nSMase1 facilitating hydrolysis of SM found in the ER/Golgi and nSMase2 within multilamellar bodies in cytosol, in the cytosolic leaflet of the plasma membrane, and in the nuclear envelope (31). SMases enable rapid increase in Cer levels in multiple cellular compartments, making them indispensable in both intracellular signaling and modifying membrane microdomains, such as rafts.

The breakdown of complex sphingolipids by different hydrolases in the lysosomal and late endosomal compartments constitutes the third pathway for Cer generation, the so-called salvage pathway (Fig. 2) (32). Cer cannot leave this compartment, but once hydrolyzed by ceramidases (CDases), the resulting Sph can be released and recycled in the ER where further reacylation by CerS regenerates Cer (32, 33). Hence, CDases are crucial controllers of the interconversion of Cer and Sph. At least five CDases have been identified, with different optimal pH levels and localizations in cellular compartments such as lysosomes, ER, Golgi, and the plasma membrane (34). The reverse action of neutral CDase can participate in Cer formation from Sph and acyl-CoA in the mitochondria (35). Notably, mitochondria house most of the enzymes involved in sphingolipid metabolism; mitochondrial CerS, SMase, and neutral CDase give rise to a local Cer pool (36, 37, 38, 39, 40), which regulates diverse cell death mechanisms, as discussed below.

Sph can also be phosphorylated by two distinct Sph kinases (SphKs), to form S1P (41), which in turn can be dephosphorylated by S1P phosphatase to regenerate Sph (Fig. 2). Interestingly, S1P can also provide the sole escape from the intricate sphingolipid metabolic pathways; S1P can be irreversibly degraded to ethanolamine 1-phosphate and hexadecenal in a reaction catalyzed by S1P lyase, the only irreversible reaction in this pathway that does not render a sphingolipid metabolic intermediate (42).

For further details on sphingolipid structure and metabolism, readers are referred to excellent reviews that extensively cover these issues (32, 33, 43, 44).

## The sphingolipid rheostat: at the crossroads between cellular survival or death

By the mid-1990s, it was clearly established that Cer and Sph regulated the induction of cell cycle arrest and cell death, whereas S1P modulated the opposite processes, enhancing proliferation and promoting cell survival and differentiation. Overwhelming evidence demonstrated a rapid and effective interconversion between these sphingolipids (Fig. 2), arising from the modulations in the activity and levels of the enzymes involved. These modulations, in turn, occur due to changes in multiple intracellular cues, resulting from the interaction of the cells with their environment. This led to the proposal that the levels of Cer and S1P provide an effective tool to monitor intracellular conditions and rapidly respond to changes in the environment, as fluctuations in their levels would activate numerous signaling pathways that control cell fate. This concept, later denominated “the sphingolipid rheostat” (5, 6), has been supported by numerous reports, and it now provides the basis for understanding the crucial roles of sphingolipids as modulators of life or death in the cell.

Later findings have extended our knowledge on the signaling pathways and molecular actors, such as C1P and Sph, participating in the sphingolipid metabolic cycle and have contributed to our understanding of their signaling capacity and extraordinary complexity. This amplified sphingolipid rheostat has been confirmed to be involved not only in normal cell physiology but also in numerous pathologies (15, 45, 46, 47, 48, 49). Cumulative evidence supports its participation in diseases affecting the retina (10, 11, 12, 50), thus providing new clues in the quest for innovative treatments for these pathologies.

## Main sphingolipids involved in retinal diseases

Individually, sphingolipids are minor components of the retinal lipid pool; however, they collectively add up to 11–13 (mole) percent of lipids in rat and bovine retinas (51). SM is the most abundant (2.4–2.5% of total retinal lipids), and accounts for 80% of the sphingolipids analyzed in mouse retinas (52). Cer is the second, amounting to around 11%. GlcCer and GalCer together represent around 4%, and Sph accounts for 0.45% of total sphingolipids (52). Most of these sphingolipids have long and very long-chain saturated fatty acids, with 16:0 and 18:0 being the major ones. Notably, they just have 2–3% DHA (22:6 n-3) and completely lack very long-chain PUFAs over 24 carbons, contrasting with the high levels of PUFA usually found in retinal lipids (51, 53).

This sphingolipid profile and the enzymes involved in their metabolism are altered in retinal pathologies, as will be discussed later. We will first briefly describe the characteristics and functions of the sphingolipids most affected in these pathologies for a better understanding of their impact.

## Cer: the death orchestrator

Cer has been a prime suspect in the cellular “crime scene” ever since its key roles in controlling cell death and growth arrest were uncovered over four decades ago. This bioactive sphingolipid is involved in senescence, inhibition of cell proliferation, inflammation, and in the induction of several pathways of cell death, including apoptosis, autophagy, and Parthanatos (15, 49, 54, 55). Cer-mediated cell death is frequently associated with mitochondrial dysfunction (56, 57, 58, 59).

In understanding the complexity of Cer synthesis, it is crucial to note that its high hydrophobicity keeps Cer in the membrane in which it has been synthesized, except when carried away through a specific transport mechanism. Although Cer was initially considered a homogeneous sphingolipid class, a “many Cers” paradigm has evolved with the isolation of over 200 distinct mammalian Cers resulting from different combinations of enzymes localized to different cell compartments (43). This “compartment-specific” synthesis model provides a basis for understanding the different functions or mechanisms triggered by Cer in different cellular locations, highlighting the relevance of the specific enzymes involved in Cer-mediated actions. Cer fatty acyl chains are vital for these effects, supporting the critical role of CerSs, which have a distinct selectivity for specific fatty acids. For example, C16:0-Cer participates in the induction of cell death (60, 61); low levels of CerS6 in colon cancer cells are sufficient to cause an ineffective C16-Cer response to tumor necrosis factor-related apoptosis-inducing ligand (TRAIL) induction of apoptosis, which is restored by expressing this enzyme (62).

The fact that its highly hydrophobic properties confine Cer to cell membranes does not diminish its ability to regulate cellular processes. In fact, it contributes to Cer’s diverse range of actions, which include modifying membrane properties, forming channels, and acting as an intracellular messenger. The biophysical properties of Cer are critical for its unique interactions with other membrane components and the modulation of membrane characteristics, leading to the reorganization of membranes and rafts. The ability of Cer to self-associate promotes the formation of small highly-ordered Cer-enriched microdomains, which can spontaneously fuse upon increases in Cer levels resulting from SMase activation, forming macrodomains that serve as signaling platforms by selective trapping of proteins. This allows for segregation, interaction, and oligomerization of proteins such as cytokines and death receptors, leading to the activation of signaling pathways such as those triggered by the proapoptotic protein Bax (63, 64, 65, 66, 67). Moreover, Cer increase has been shown to displace cholesterol and caveolin from membrane domains (68), thus modifying their biophysical properties. Hence, Cer-enriched domains differ both structurally and functionally from traditional membrane rafts and caveolae.

Cer is essential for the formation and/or secretion of exosomes by facilitating or inducing membrane curvature (69, 70). Cer enrichment in exosomes has led to the proposal that it may participate in the transmission of “mobile rafts” from donor to recipient cells (70). The increase in Cer levels in mitochondria is decisive for the induction of cell death. Extensive evidence supports that Cer can self-assemble to form channels composed of many Cer monomers, which are able to translocate proteins and have been associated with Cer’s ability to induce both apoptosis and necrosis. These channels have been shown to promote the release of cytochrome c from mitochondria (61, 71, 72, 73). Interaction between Cer and the Bcl-2 family of proteins is crucial for controlling mitochondrial outer membrane permeability, a central step in apoptosis signaling. Formation of Cer channels and release of cytochrome c are inhibited by Bcl-2 anti-apoptotic proteins, such as Bcl-XL and Bcl-2; in turn, Cer has been shown to promote Bax oligomerization and pore formation and/or to act synergistically with Bax and Bak, possibly by forming hybrid channels (74). In addition, Cer inhibits the respiratory chain and stimulates ROS overproduction (75, 76).

Cer also acts as a versatile second messenger. Its capacity to activate protein phosphatases of the PP1 and PP2A families grants Cer a role in controlling the cellular phospho-proteome, including the activity of protein kinase C (PKC), Akt, and ezrin (33, 77, 78, 79). PP2A can dephosphorylate and inactivate anti-apoptotic proteins such as Bcl-2, AKT, and c-Myc (80, 81). Cer targets depend on its site of generation; thus, lysosome-generated Cer triggers cathepsin B activation, whereas mitochondrial Cer induces Bax-dependent apoptosis (82, 83).

The most investigated function of Cer is its role in the induction of cell death. A diverse array of cell stressors such as hypoxia, DNA damage, growth factor withdrawal, ionizing radiation, oxidative damage, or death factors increase the levels of Cer, which then triggers either the intrinsic or the extrinsic apoptotic pathways (78, 84, 85). Cer also plays a key role in the regulation of both survival and lethal autophagy, acting at steps ranging from initiation to autophagosome formation (86). C18-Cer, generated by CerS1, induces selective mitochondrial autophagy, also known as mitophagy (87). Although mitophagy can play a role in either survival or cell death, mitochondria-generated Cer triggers lethal mitophagy, particularly by binding to LC3II-containing autophagosomes (87, 88). Cer has also been shown to activate necroptosis, which is triggered by high levels of C16:0-Cer (89). Recent evidence also establishes that Cer induces Parthanatos, causing neuroblastoma and photoreceptor cell death (55, 90).

Cer is now known to participate in the progression of multiple pathologies, including inflammation, metabolic syndromes such as obesity and insulin resistance, vascular diseases such as ischemic injury and atherosclerosis, cancer, and neurological disorders (91, 92, 93). Recent cardiovascular trials highlight a novel role for Cer as a biomarker of cardiovascular diseases, associating plasma Cer levels and distinct serum Cers with the risk of major cardiovascular events (94, 95). Knowledge of its involvement and roles in these diseases is constantly expanding, and excellent collation on its pathophysiological impact can be found in recent reviews (33, 96, 97).

The observation that Cer accumulates in the retinas of patients with Farber disease, which primarily affects ganglion cells and is associated with visual dysfunction, suggests its involvement in retinal pathologies (98). The first direct evidence of this involvement came from the observation that transgenic expression of a neutral CDase prevents retinal degeneration in *Drosophila* phototransduction mutants by decreasing Cer levels (99, 100). Since then, extensive work has shown its contribution to retinal physiology and pathology, as we will analyze in this review.

## S1P: the good, the bad, and the ugly combined?

The last thirty years have seen the emergence of another star in the world of bioactive lipids: S1P. S1P plays an incredibly diverse array of vital functions in virtually every cell of every organism, having both beneficial and deleterious roles. The basis for this dichotomic behavior lies in the ability of S1P to regulate several cellular processes such as proliferation, survival, differentiation, and cell movement, as well as more complex responses such as vascular development, inflammation, and immune cell trafficking (101, 102).

As described, S1P is a molecular intermediate in the complex sphingolipid network that can easily interconvert with its precursor, Sph, and be further metabolized to Cer. Because S1P displays opposing cellular roles to both Sph and Cer, the balance of the relative levels of these sphingolipids constitutes the “sphingolipid rheostat”, which ultimately determines cell fate (5). S1P is synthesized through the phosphorylation of Sph by two SphKs, SphK1 and SphK2, which not only differ in their cellular localization but also generate S1P with distinct and at times opposing functions (103). SphK1 resides in the cytosol and is preferentially located near the plasma membrane. The S1P it produces acts as a second messenger or is secreted to become an extracellular ligand. SphK2 is localized in the nucleus and mitochondria, and the S1P it generates functions as a histone deacetylase inhibitor, thus regulating gene expression (104). High plasma levels of S1P have been proposed to depend mainly upon its release by vascular endothelial cells and red blood cells (105, 106, 107). Circulating S1P is transported bound to plasma protein chaperones, mainly HDL and albumin, but also in smaller amounts by other lipoproteins (107). Multiple cell stimuli promote intracellular generation of S1P, which then acts as an extracellular ligand. Following export to the extracellular milieu by different cell transporters such as Spinster 2 (Spns2) (108), ABCA1 (109), ABCC1 (110), and ABCG2 (111), S1P then binds to and activates a family of five S1P receptors, termed S1PR1–5, in an autocrine/paracrine fashion termed “inside-out” signaling (112). These receptors belong to the superfamily of G protein-coupled membrane receptors that are ubiquitously expressed and activate different G proteins to regulate multiple downstream effectors including PI3K, adenylate cyclase, protein kinase-C, phospholipase C, and intracellular calcium (113, 114). To add further complexity to its signaling pathways, S1P has been proposed to upregulate the transcription of SphK1, activating an “outside-in” S1P/SphK1 signaling axis (115). These intricate signaling networks allow S1P to trigger a myriad of cellular responses resulting from diverse combinations of cellular localization, receptors, and downstream signaling cascades activated by S1P. Therefore, it is not surprising that S1P activation of S1PRs is not only involved in many pathophysiological processes by regulating proliferation, differentiation, cell migration, cellular barrier integrity, angiogenesis, and immunity, but also contributes to disease processes such as inflammation, atherosclerosis, fibrosis, and neoplasia (116, 117, 118). For instance, S1P activation of S1PR1 is critical for the progression of autoimmune diseases (117).

In the retina, S1P has both beneficial and detrimental properties. On the one hand, S1P promotes normal retinal morphogenesis (119, 120) and facilitates signaling in the inner retinal cells (121). S1P signaling through S1PR1–3 is essential for the adequate development of retinal vasculature; the coordinate signaling of retinal endothelial S1P and vascular endothelial growth factor (VEGF) results in the formation of the trophic factor gradient essential for the growth and maturation of retinal vasculature (107, 122, 123). S1P induces the proliferation and later differentiation of retinal progenitors into photoreceptors (124) and mediates photoreceptor survival upon oxidative damage (11, 124, 125); although this supports a role for S1P during retina development, this remains to be confirmed. Moreover, several photoreceptor trophic factors such as glial-derived neurotrophic factor, DHA (126), and nerve growth factor (NGF) (127) stimulate the S1P/SphK1 axis to enhance the levels of S1P and thus elicit their beneficial roles. On the other hand, S1P triggers threatening processes in cells with crucial support functions in the retina, i.e., the retinal pigment epithelium (RPE) and Müller glial cells. They include secretion of pro-inflammatory cytokines, proliferation, trans-differentiation, and migration (128, 129, 130, 131), all of which alter the retinal structure and may contribute to visual dysfunction. We will later discuss the role of S1P, among other sphingolipids, in the development of retinal pathologies.

## C1P: a complementing performer

First identified in the brain in the late eighties, C1P is now an established bioactive sphingolipid involved in numerous cellular processes such as cell proliferation, survival, and growth, and chemotaxis. To date, CerK is the only enzyme known to catalyze C1P synthesis in mammals (132). This enzyme is most abundant in the Golgi, though it is also expressed in the cytosol, plasma membrane, nucleus, and perinuclear membranes (133). C1P is present both intra- and extracellularly. Once synthesized, it is transported through a specific Cer phosphate transfer protein (CPTP) to the plasma membrane (134). Although it is not highly permeable, C1P can cross the cell membrane to be released to the extracellular milieu, where it is found in concentrations as high as 20 μM (22, 135, 136). Existence of a C1P-specific transporter and its secretion in vesicles have been reported (134, 137). C1P is a second messenger as well as an extracellular ligand, activating multiple signaling pathways including PI3K, ERK/MAPK, Jun N-terminal kinase (JNK), cytosolic phospholipase A2, NF-κB, and glycogen synthase kinase 3 (GSK3) (138). As an extracellular ligand, C1P interacts with a G protein-coupled receptor, which is not yet fully characterized, although it is known to differ from S1PRs (139).

Recent findings indicate that C1P promotes cell migration (140, 141), proliferation (142, 143, 144), and survival, as C1P is also known to be antiapoptotic (139, 145). These actions make C1P a relevant signal transducer for cancer progression (143, 146). C1P can act as a pro- or anti-inflammatory signal, depending on the cell type (133, 147, 148), and also has neuroprotective effects in the nervous system (149, 150) contributing to neurotransmitter release (151).

In the retina, C1P functions are still elusive. CerK is highly expressed in the retina (152), and it is present in the RPE cells (153). CerK is critical for controlling C1P levels in this tissue, as it is markedly reduced in *Cerk*^−/−^ mouse retinas (154). C1P promotes the proliferation of photoreceptor progenitors and their differentiation as photoreceptors in vitro (155). C1P also promotes photoreceptor survival through the preservation of their mitochondrial potential (155) and probably also by preventing the accumulation of Cer, a mechanism already observed in macrophages (139, 145). We will discuss the role of C1P in the development of retinal pathologies in a later part of this review.

## Other sphingolipid players in retinal pathologies

### Sph

Along with Cer, Sph is an endogenous mediator of apoptosis and its addition inhibits proliferation and/or induces apoptosis in many cell types in vitro (156). Different apoptotic inducers, such as oxidative stress, chemotherapy, environmental stress, and tumor necrosis factor α (TNF-α), rapidly increase the levels of both Cer and Sph, which then induce cell cycle arrest, senescence, or apoptosis (96, 156, 157, 158, 159). Usually, Cer upsurge precedes that of Sph, implying that Sph accumulation results mainly from the deacylation of Cer, catalyzed by CDases (157, 159). The fact that Sph can be either rapidly recycled to regenerate Cer or phosphorylated by SphKs to render S1P (Fig. 2) has complicated ascertaining Sph’s effects. However, apoptosis in thymocytes and 3T3/A31 is drastically reduced by inhibiting Sph synthesis (160, 161). Sph itself induces apoptosis in cells under conditions where Cer is unable to do so and when Sph conversion to Cer is blocked (157, 162). These findings have contributed in establishing Sph as a bona fide second messenger, whose increase is triggered by diverse apoptotic stimuli to induce cell death.

Sph modulates the functions of several signaling molecules to promote cell death. In addition to PKC, Sph activates protein kinase A (163) and inhibits calmodulin-dependent kinases (9). Sph induces apoptosis through the generation of ROS and downregulation of Bcl-2, with the consequent activation of the mitochondrial pathway, cytochrome c release, and caspase-3 activation (126, 157, 162). This also involves downregulation of the pro-survival signaling through Akt signaling together with increased phosphorylation of 14-3-3 protein and its consequent inability to sequester BAD/Bax (161). Mitochondrial dysfunction is apparently instrumental in Sph-induced cell death, because preventing it by overexpressing Bcl-xL impedes cell death, even upon increased levels of Sph (164). Mitochondrial accumulation of Sph impairs the electron transport chain and has been proposed to be critical for brain injury after trauma (165).

In the retina, both enhanced endogenous synthesis and exogenous addition of Sph promote the death of photoreceptors and amacrine neurons (126). Oxidative stress increases the synthesis of Sph, leading to photoreceptor death, and this death is prevented by inhibition of alkaline CDase. Sph promotes ROS formation, mitochondrial permeabilization, and cytochrome c release leading to photoreceptor apoptosis. Notably, DHA protects photoreceptors by increasing SphK1 expression and translocation to the plasma membrane, suggesting that the increased generation of S1P and/or the consequent decrease in Sph levels prevent their death (126). In contrast, overexpression of acid CDase in a human RPE cell line, ARPE-19, increases Sph levels and protects these cells from oxidative damage with no visible accumulation of S1P (166). Although further research is required to establish Sph’s effects in different retinal cell types, the existing data have cemented Sph and Cer as crucial mediators in the onset of photoreceptor death and support the hypothesis that modulation of the sphingolipid pathways may provide powerful tools for treating neurodegenerative diseases of the retina.

### GlcCer and LacCer

The complex sphingolipid metabolic routes provide alternative pathways to prevent the increase in Cer by converting it to glycosyl Cers and thus avoid the effects of its accumulation. Many different molecules regulate the expression and activity of GlcCer synthases (GCSs), which catalyze GlcCer synthesis from Cer (167). GlcCer is found in multiple animal tissues, such as spleen, skin, erythrocytes, and the nervous system, and has often ambiguous roles in mammalian cells. It is essential for preserving the water permeability barrier of skin, and its levels in tissues are affected in skin disorders, diabetes, cardiovascular diseases, and cancer (168). Its formation serves as an escape route preventing Cer accretion and the consequent induction of cell death and has been associated to drug resistance in several cancers (169). GCS expression is linked to poor prognosis in certain cancer patients (170), whereas its inhibition attenuates resistance to chemotherapy in different tumor cells (171, 172). GlcCer is involved in cell proliferation, differentiation, oncogenic transformation, and tumor metastasis (167, 173). It has been shown to inhibit LPS-induced inflammation in macrophages by blocking nuclear translocation of NF-κB (174), and also have immunostimulatory functions, acting as a ligand for lectin receptors sensing damaged cells (175).

In the eye, GlcCer increases in the retinas of diabetic rats and preventing this increase augments insulin sensitivity and is neuroprotective, linking GlcCer accumulation to the pathogenesis of diabetic retinopathy (DR) (176). GlcCer also accumulates in retinas of patients with Gaucher’s disease, resulting in visual loss (177). Interestingly, inhibiting GlcCer synthesis in photoreceptors abrogates the protective effect of DHA upon oxidative stress and Cer increase (178). Hence, GlcCer might be either protective or deleterious in the retina in a context- and concentration-dependent manner.

In the Golgi, GlcCer can be converted by LacCer synthases to LacCer, which has a pivotal role in the synthesis of most major glycosphingolipids. Cellular functions of LacCer are still ill-defined. It has been proposed that several molecules, such as growth factors, pro-inflammatory cytokines, and modified LDL increase LacCer levels, activating multiple pathways that contribute to cell proliferation, adhesion, migration, angiogenesis, and apoptosis (179, 180). LacCer is thought to mediate the attachment of many pathogens and may participate in the innate response to them, especially on nonimmune cells (181, 182). It is enriched in the plasma membrane of neutrophils, promoting their migration and phagocytosis, and mediating innate immune functions (183). An accumulation of LacCer has been linked to pathogenic alterations in diseases affecting different organs. In diabetic mice, an increased Cer flux leads to elevated levels of LacCer in cardiac tissue and contributes to mitochondrial dysfunction (184). Furthermore, oxidative stress leads to LacCer accumulation in retinal endothelial cells, suggesting its possible role in inflammatory eye diseases (10).

## Sphingolipids in retinal pathogenesis

During the last decade, evidence has been acquired that supports the relevance and association of sphingolipids in multiple retinal diseases (Table 1). In the next part of the review we will focus on the involvement of sphingolipids like Cer, Sph, S1P, C1P, and glycosylceramides (HexCer and LacCer) in multiple retinal pathologies.

**Table 1:** Association of bioactive sphingolipids with different retinal diseases/pathologies

### Age-related macular degeneration: watching sphingolipids at work?

Age-related macular degeneration (AMD) is a degenerative disease of the macula that accounts for approximately half of all legal blindness in industrialized countries (185). Among the two subtypes, nonexudative or atrophic AMD (also called dry AMD) is a broad designation, encompassing all forms that do not result in neovascularization. This includes early and intermediate forms of AMD, as well as the advanced form of dry AMD known as geographic atrophy (GA). Atrophic AMD has a relatively poorly understood etiology and no effective treatment. It involves the formation of drusen between the RPE and the Bruch’s membrane, leading to slow but increasing RPE and photoreceptor degeneration and progressive GA (186). On the other hand, in exudative or neovascular AMD (also known as wet AMD), vision loss is due to abnormal choroidal neovascularization. It is characterized by overproduction of VEGF in the RPE, responsible for breakdown of the blood-retinal barrier and choroidal/subretinal neovascularization (187). The proliferation of abnormal blood vessels in the retina, which are more fragile than typical blood vessels, leads to hemorrhage, causing macular scarring and edema, which is the major cause of vision loss in exudative AMD (188). However, degeneration of the RPE cells and subsequent photoreceptor death leading to loss of central vision is the hallmark of both forms of AMD. Several studies have proposed a connection between inflammatory mechanisms and AMD pathology (189, 190, 191). Subretinal drusen contain a variety of potentially harmful constituents such as lipids, RPE-derived cellular debris, oxidation byproducts, and inflammatory factors including complement components and immunoglobulins (192, 193, 194, 195). Complement factor H (CFH), a major inhibitor of the complement pathway, is synthesized by RPE cells and accumulates within drusen; the variant harboring a point mutation Y402H in the *CFH* gene has been identified as a major risk factor for the development of AMD (196, 197, 198, 199). Further associations have been identified between AMD and several complement pathway-associated genes: complement factor B, complement factor H-related 1 and 3, and complement components 2 and 3 (200). Interestingly, a recent study showed that the Y402H variant in the *CFH* gene influences the association of high serum Cer levels with GA, and high levels of HexCer in the serum of patients with choroidal neovascularization and GA (201).

Increasing evidence supports altered sphingolipid levels contributing to AMD pathology (153). Degeneration and death of photoreceptor and RPE cells is the ultimate cause of blindness in AMD, and Cer-induced inflammation and apoptosis have been linked to degeneration of both cell types in different models of AMD and other ocular degenerative diseases (153). Chen et al. (202) have shown that the increase in Cer levels by de novo biosynthesis mediates photoreceptor apoptosis in a rat model of light-induced retinal degeneration, a pathology with significant overlap with human atrophic AMD, whereas inhibiting Cer production protects the retina against light stress. Elevated Cer levels trigger photoreceptor death in different in vitro models of retinal degeneration. Oxidative stress increases de novo synthesis of Cer in cultured rat retinal neurons and induces photoreceptor death by affecting mitochondrial function, whereas lowering Cer levels by inhibiting its synthesis or promoting its glycosylation to GlcCer prevents photoreceptor death (203). Oxidative stress also induces apoptosis of the 661W photoreceptor-like cell line through the activation of aSMase and subsequent Cer increase, which activates the mitochondrial pathway of apoptosis, the caspase cascade, and also the calpain- and cathepsin-mediated death pathways. Again, inhibiting aSMase-dependent Cer synthesis prevented cell death (204). Cer has recently been shown to induce cell death in cultured photoreceptors through the Parthanatos death pathway, involving activation of poly-ADP ribose polymerase 1 (PARP-1) and calpains (55). As a whole, these studies clearly establish Cer as a master controller of the cell death decision in photoreceptor cells independent of its biosynthetic pathway. The increase in Cer levels triggers cell death through a diversity of pathways, suggesting that the biosynthetic pathway and the cell death routines may be context- and cell type-dependent.

Cer has also been shown to be a crucial player in the induction of RPE cell death. Cer addition to human cultured RPE cells increases the levels of ROS, promoting mitochondrial permeabilization and caspase-3 activation, leading to RPE cell apoptosis (205). Oxidative stress has been shown to induce Cer synthesis and promote apoptosis of human cultured RPE cells (206) and also induces cell death in ARPE-19 cells, increasing Cer and HexCer levels. Conversely, overexpression of acid CDase diminishes Cer levels by hydrolyzing it to Sph, and partially decreases cell death, probably by transforming at least part of the generated Sph into S1P (166). Conversely, overexpression of nSMase, which increases Cer generation, promotes ARPE-19 cell death (153). Cer has been implicated in AMD-related RPE degeneration, wherein activation of aSMase results in RPE autophagy dysfunction, complement regulatory protein recycling, endosome biogenesis, and complement activation (207, 208, 209, 210). These data highlight the involvement of Cer in the degeneration and death of photoreceptors and RPE cells; because these are critical events for AMD onset and progression, Cer may have a role in triggering this disease and controlling its metabolism may provide a therapeutic strategy for this disease.

Other sphingolipids may also be involved in AMD progression. Sph has also been implicated in photoreceptor death; its addition induces photoreceptor apoptosis, increasing ROS production and promoting cytochrome c release from mitochondria (126). The pro-inflammatory state of RPE together with its release of proangiogenic factors is known to contribute to AMD development. Mounting evidence supports a role for S1P, a well-known mediator of inflammation and neovascularization, in these processes. Recent work has shown that S1P promotes the secretion of inflammatory cytokines by ARPE-19 cells (211). In addition, S1PR2 deficient mice show marked downregulation of laser-induced choroidal neovascularization (212), a hallmark of wet AMD. This neovascularization is also significantly reduced when S1P action is blocked with sonepcizumab, a humanized monoclonal antibody against S1P (213). Puzzlingly, S1P has been shown to prevent neuronal death in different models of retinal injuries. S1P also promotes differentiation and survival of cultured photoreceptors (8). The expression of SphK1, S1PR2, and S1PR3 rapidly increases in a rat model of light-induced retinal degeneration, suggesting a function for S1P signaling in light stress responses in the retina (214). Due to the multiple processes it modulates, S1P may have opposing functions in the development of AMD, on the one hand promoting survival of photoreceptors and on the other hand contributing to the progression of inflammation and neovascularization. Further research is needed for establishing the functions of S1P and uncovering the signaling mechanisms it triggers.

In conclusion, these findings establish that sphingolipids play important roles in central features contributing to AMD pathology by regulating retinal cell death, inflammation, and neovascularization and may therefore be involved in its onset and/or progression. Controlling their metabolism and the intracellular pathways they activate may provide novel targets and therapeutic strategies for treating this devastating disease.

### Retinal inflammation and uveitis: are sphingolipids critical regulators?

Uveitis is an autoimmune eye disease characterized by inflammation of the uvea, specifically in the middle layer of the eye consisting of the anterior uvea (iris and ciliary body) and the posterior uvea (choroid) (10). Common symptoms of anterior uveitis include pain, erythema, and photophobia, while intermediate and posterior uveitis results in visual deficits (50) leading to loss of vision of approximately 30,000 people annually in the United States (215). The inflammation resulting in uveitis can arise from a number of diseases ranging from a viral infection to ocular trauma and systemic disease (216). It can cause severe damage to the retina, optic nerve, and vitreous, often leading to complications such as macular edema, development of cataracts, and glaucoma (217). The inflammation associated with uveitis is due to infiltration of both innate and adaptive immune cells (218). The characteristic inflammatory reaction involves CD4+ T-cells activated against retinal cells, as has been shown in an animal model of experimental autoimmune uveoretinitis (EAU) (219). Th17 and Th1 T-cells also play a significant role in the inflammatory mechanism of uveitis. The helper T-cells recruit different effector immune cells, including neutrophils and monocytes, responsible for tissue destruction, with pro-inflammatory cytokines playing a major role (220).

Uveitis can arise from inflammation in the eye itself or it can be a manifestation of diseases affecting multiple organs like systemic sarcoidosis (221), where about 70% of the cases result in anterior granulomatous uveitis (222). Uveitis can also be a complication of the autoimmune disease multiple sclerosis, affecting between 1% and 10% of patients with this disease (223). Multiple sclerosis is characterized by immune-mediated demyelination and inflammation of the CNS, and both the innate and adaptive immune systems are known to be involved in its development, recruiting microglia, activated macrophages, and both B and T lymphocytes (224). The cause of uveitis in patients with multiple sclerosis is unknown, but myelin basic protein and myelin oligodendrocyte glycoprotein have been shown to promote autoimmune uveitis in animal models (225). An autoimmune reaction resulting from sensitization of the immune system to antigens expressed in the CNS has been proposed as a trigger. Because nerve and ocular tissues derive from the same embryonic cells, multiple sclerosis and uveitis may share some etiologic factors (226).

Recent evidence suggests a role of sphingolipids in autoimmune eye diseases such as uveitis. Fingolimod (FTY720), a Food and Drug Administration-approved therapeutic drug for multiple sclerosis, has been found to be effective in a rat model of experimental autoimmune uveitis (227). FTY720 is a structural analog of Sph and has different targets in the complex sphingolipid metabolic network. FTY720 phosphorylation by SphK2 results in its active form, FTY720-phosphate, which mimics S1P and is a functional antagonist of almost all S1PRs, with the exception of S1PR2 (228). FTY720-phosphate binds to S1PR1, preventing its activation by S1P, and promotes its internalization and degradation, thus blocking the egress of lymphocytes from the lymph nodes (229). FTY720 also blocks de novo Cer synthesis by inhibiting CerSs (230). This grants FTY720 the ability to modulate both Cer synthesis and S1P signaling, thus affecting the “sphingolipid rheostat” and, consequently, sphingolipid signaling (231). In patients with Vogt-Koyanagi-Harada uveitis, T cell clones from aqueous humor (AH) or peripheral blood mononuclear cells produce high levels of pro-inflammatory cytokines IL-6, IL-8, and IFN-γ; treatment with FTY720 suppresses T cell production of granulocyte monocyte colony stimulating factor (232). FTY720 has been shown to suppress both the incidence and intensity of inflammation in a dose-dependent manner in an animal model of EAU (233), and to prevent inflammatory cells from infiltrating the retina, when administered prior to the onset of EAU (227, 234, 235). FTY720’s effects may result from its antagonizing S1P signaling through S1PR1, suggesting that S1P is involved in promoting inflammation and migration in EAU. A similar effect has been reported in clinical cases of uveitis (232). Although the molecular mechanisms of sphingolipid regulation of cytokine production by inflammatory cells remain to be elucidated, both Cer and S1P are known to modulate inflammation, which is crucial for the pathogenesis of inflammatory neural and ocular diseases (236, 237, 238). The ability of FTY720 to modify the course of uveitis in humans and in animal models together with its capability to modulate Cer and S1P synthesis and signaling suggest a role for sphingolipids in inflammation and lymphocyte migration in this disease.

Recent lipidomics data show that total sphingolipid levels increase during the acute inflammatory stage in a rat model of endotoxin-induced uveitis; enhanced levels of C12-C1P, C16-C1P, and C24-C1P are present in the retina, while the levels of C24:0 and C24:1 Cer and C24:0 SM are augmented in the AH. Furthermore, endotoxin-induced uveitis rats have increased levels of pro-inflammatory cytokines IL-6 and TNF-α in the AH, and of pro-inflammatory transcription factor NF-kB in the retina (239). These observations suggest a role for C1P and Cer in the infiltration of innate and adaptive immune cells leading to inflammation in this animal model of uveitis. Patients with Gaucher disease have shown elevated levels of GlcCer, resulting in vitreous opacity and subsequent infiltration of macrophages, suggesting the involvement of Cer and GlcCer in certain forms of uveitis (240, 241).

Although further research is required to establish the involvement of sphingolipids in the onset or progression of uveitis, their increase in inflammatory ocular diseases and the effectiveness of FTY720 in limiting the intensity of uveitis in animal models suggest a role for these lipids in this pathology. It also strongly supports FTY720’s potential for the treatment of inflammatory ocular diseases and underscores the relevance of the identification of novel molecular targets within the sphingolipid metabolic pathways for future drug development.

### Glaucoma: sphingolipids as the emerging players?

Glaucoma is a family of ocular pathologies traditionally defined by optic nerve damage resulting from elevated intraocular pressure (IOP). It is one of the leading causes of irreversible blindness affecting approximately 80 million people worldwide (242, 243). There are different types of glaucoma; and although multiple factors contribute to the elevation of IOP, progressive optic nerve degeneration and retinal ganglion cell death are common features. Angle-closure glaucoma is characterized by narrowing or complete closure of the anterior chamber angle, which prevents the drainage of AH, resulting in IOP elevation leading to optic nerve damage. It may result from anatomical predispositions such as defects in the iris or lens (primary angle-closure glaucoma) or from a secondary process such as neovascularization or inflammation (as in secondary angle-closure glaucoma) (244). In open-angle glaucoma (OAG), the blockage of the trabecular meshwork increases aqueous outflow resistance, also leading to gradual IOP elevation and subsequent optic nerve damage (245). In contrast, normal tension glaucoma is characterized by normal or low IOP along with ganglion cell death, optic nerve degeneration, and visual field defects similar to those of other types of glaucoma involving IOP elevation (246, 247). Elevation of IOP can cause mechanical stress and strain on the posterior structures of the eye, particularly the lamina cribrosa and adjacent tissues (248, 249). Being structurally weaker than the much thicker and denser sclera, the lamina cribrosa is more sensitive to these changes, which may cause its compression, deformation, and remodeling. This provokes mechanical axonal damage and disruption of axonal transport, thus interrupting the delivery of essential trophic factors to retinal ganglion cells (250, 251). Disrupted axonal transport occurs early in the pathogenesis of experimental systems of glaucoma (249). The occurrence of mitochondrial dysfunction in retinal ganglion cells and astrocytes has also been suggested during periods of high IOP leading to energetic stress (252). The primary neural pathological processes may also lead to secondary degeneration of other retinal neuronal cells in the central visual pathway by altering their environment and increasing their susceptibility to damage (244, 253).

Factors such as impaired microcirculation, altered immunity, excitotoxicity, oxidative stress, and inflammation may also play a role in the pathogenesis of glaucoma. Neuroinflammatory responses during early stages of glaucoma are mediated by astrocytes, resident microglia, and other monocyte-derived cells in the optic nerve head. Microglial reactivity was involved in early alterations in axonal transport in a rat model of glaucoma (254), and proteomic analysis revealed upregulation of TLR signaling along with increased expression of TLRs on both microglia and astrocytes in human glaucomatous retinas (255). Similarly, in the DBA/2J mouse model of glaucoma, upregulation of 11 out of 13 TLRs in the optic nerve head led to activation of pro-inflammatory cytokines (256, 257). This suggests the involvement of the inflammatory response in the pathogenesis of glaucoma.

Mounting evidence supports a role for bioactive sphingolipids, key players in cellular inflammation, in the pathobiology of glaucoma. Genetic and genome-wide association studies have suggested a connection between impaired sphingolipid metabolism and glaucoma (258, 259), and lipidomic studies have identified several unique species of Sph, Cer, and SM in human glaucomatous AH (260). The lipidomic profile of AH from OAG patients shows an increase in SM species along with increased activity of SMS and decreased activity of aSMase (261). SMs and phosphocholine species have been linked to the physiopathology of OAG by modulating trabecular meshwork resistance and AH outflow (260). S1PR2 has been identified as a mediator of trabecular meshwork contractility, affecting aqueous outflow and having a potential role in glaucoma pathogenesis (262, 263). In addition, metabolomic profiling of plasma from primary OAG (POAG) patients showed high levels of Sph and sphinganine and low levels of S1P, further emphasizing the alteration of sphingolipid metabolism in this disease (264). Analysis of the sphingolipid changes in human optic nerves from POAG patients revealed increased levels of glucosylsphingosine and of lysosomal and nonlysosomal acid *N*-acyl-sphingosine amidohydrolases (ASAHs: ASAH1 and ASAH2), consistent with increased conversion and accumulation of glucosylsphingosine, together with normal levels of Cer and SM (265). The study supports the hypothesis that lysosomal abnormalities in glaucoma also occur in the posterior ocular tissues, including the optic nerve, and provides the first step in the search for the precise region and cells contributing to these changes in POAG (265). A recent study from the same group has identified low levels of several sphingolipids in the AH and trabecular meshwork in POAG patients and has shown that these sphingolipids reduce IOP in most mouse ocular hypertensive models (266). As a whole, these findings demonstrate that sphingolipid composition and metabolism are altered in glaucoma and emphasize the therapeutic potential of modulating sphingolipid levels as a novel approach for the treatment of glaucoma.

Retinal ganglion cell degeneration secondary to axon insult at the optic nerve head is a key event leading to vision loss in glaucoma (267), and sphingolipids have been shown to be involved in this degeneration. GM1 gangliosides have a critical role in ganglion cell loss in the DBA/2J mouse model of glaucoma. Gangliosides are most abundant in the nervous tissue and localize in the outer leaflets of the plasma membrane, in glycolipid-enriched microdomains, or rafts, which also include GPI-linked proteins such as Thy1, glycosphingolipids, caveolin, IgE receptors, and other membrane components (268). A link between GM1 and growth factors has been shown in several neurodegenerative diseases like Huntington disease and Parkinson disease (269, 270). GM1 has been associated with NGF and brain-derived neurotrophic factor (BDNF) effects, as it forms large clusters in rafts specialized for signaling through both neurotrophic factors as well as clathrin-mediated endocytosis (271). Both NGF and BDNF play neuroprotective roles in mouse models of glaucoma (272, 273, 274). Dysfunctional retinal ganglion cells stopped expressing GM1 in an animal model of glaucoma and this decreased expression may have affected the neuroprotective role played by NGF and BDNF (275). This finding supports a role for complex sphingolipids in glaucomic degeneration and identifies GM1 as a possible therapeutic target. Further work will help us understand the relationship between GM1, growth factors, and neurodegeneration in the glaucomic retina.

Cer has also been implicated in the mechanisms of retinal ganglion cell degeneration (276, 277, 278). Cer generation by aSMase contributes to the onset of ischemic retinal injury. Robust elevations of C16-Cer, C18-Cer, and C20-Cer, along with increased aSMase activity, occur following ischemic injury in the retina, correlating with increased inflammatory signaling, decreased visual function, and neuronal degeneration in the ganglion cell layer. These alterations are reversed by treatment with the aSMase inhibitor, desipramine, or in aSMase^+/−^ mice (278). In addition, specific conditional deletion of acid CDase in mouse retinal progenitor cells causes age-related vision loss with early ganglion cell degeneration (279). These results demonstrate a role for Cer increase in ganglion cell death and support the hypothesis that conditions leading to a buildup of Cer, either resulting from increased aSMase activity and/or expression, or from acid CDase deletion, contribute to inflammatory signaling and subsequent ischemic neurodegeneration in the retina.

S1P may have dual roles in glaucoma. Expression of S1PR1 is upregulated in a chronic hypertensive glaucoma model, and antagonizing this receptor with FTY720 attenuates ganglion cell loss and preserves visual function in the inner retina (280), suggesting that exacerbated S1P signaling through S1PR1 may regulate retinal inflammation. Paradoxically, S1PR1 expression has been shown to be required for ganglion cell survival and axonal growth after an acute optic nerve injury, as silencing this expression exacerbates neuronal loss (281). These apparently contradictory results suggest that S1P may have opposite functions in acute and chronic retinal injuries, initially inducing survival but promoting inflammatory conditions at later stages of the disease.

Collectively, these studies provide evidence for a strong connection between altered sphingolipid levels and the pathophysiology of glaucoma, further supporting the relevance of modulating sphingolipid metabolism for developing new strategies to treat this disease.

### Retinitis pigmentosa: is Cer a common activator?

Retinitis pigmentosa (RP) embodies a family of retinal degenerative diseases caused by the progressive loss of photoreceptors, in which a primary degeneration of rods is usually followed by a secondary degeneration of cones (282). As the rods die, patients experience night blindness followed by concentric visual field loss; in the late stages of RP, the death of cones leads to diurnal visual impairment and central visual loss (283). RP is a leading cause of visual dysfunction, and it is estimated to affect approximately 1 in 4,000 people worldwide. It encompasses a group of inherited disorders resulting from mutations in more than 80 genes (284), most of them essential for photoreceptor function. These mutations lead to damage in retinal structure and photoreceptor function and/or viability.

Due to the highly heterogeneous nature and diversity of genetic mutations that provoke RP, specific targets for developing treatments for this disease are still lacking. As the death of photoreceptors is a hallmark of this disease, identifying pathways and mediators that induce this cell death may provide new therapeutic strategies. As stated above, modulation of sphingolipid metabolism in the retina emerges as a common target for treating several retinal degenerations, and substantial evidence points to its potential in RP therapeutics. Several studies indicate a strong association between Cer accumulation and photoreceptor demise in mouse and rat models of retinal degeneration (202, 285, 286). Pharmacological inhibition of Cer synthesis using either topical formulations or systemic or intraocular injections that lower retinal Cer levels rescues photoreceptors from apoptotic death in rat and rd10 mouse models of retinal degeneration (285, 286, 287, 288, 289). In vivo studies have shown that Cer levels double in retinas from rd10 mice during the period of maximum photoreceptor death, whereas inhibiting Cer de novo synthesis with myriocin markedly reduces the loss of photoreceptors, preserving their morphology, survival, and visual response (286, 288, 290, 291). The link between Cer increase and RP has been emphasized by the recent findings that mutations in the adiponectin receptor 1 (ADIPOR1), which has an intrinsic CDase activity, cause RP (292, 293, 294, 295). Activation of this receptor by adiponectin has been shown to enhance Cer catabolism and formation of S1P in pancreatic β cells (292). ADIPOR1 is highly enriched in photoreceptors and RPE, facilitating the uptake and retention of DHA; knocking it down in both mice and a zebrafish model leads to photoreceptor degeneration, which may result from the increase in Cer and the concomitant decrease in DHA levels, as DHA protects photoreceptors from Cer-induced death (295, 296, 297). Interestingly, another ADIPOR1 variant has been associated with AMD in the Finnish population (298). Collectively, these studies establish Cer as a central actor in the onset of photoreceptor death in RP.

Increased oxidative stress has a decisive role in inducing photoreceptor death in retinal degeneration. Notably, both in vivo and in vitro genetic mutations and oxidative stress lead to accumulation of Cers and sphingolipid metabolites that provoke photoreceptor death, which is prevented by inhibiting this accumulation (126, 203, 285). Retinas from the rhodopsin mutant retinal degeneration, P23H-1, rat model show high levels of major sphingolipid species at early stages of degeneration, including Cer, HexCer, and S1P. However, systemic treatment with FTY720 rebalances the sphingolipid profile, prevents retinal degeneration, and improves visual function (285). Photo-oxidative damage promotes Cer increase through the de novo pathway in a light-induced retinal degeneration model, and FTY720 effectively prevents this increase, protecting retinal structure and function (202). Both Cer and Sph are mediators of oxidative stress-induced death of photoreceptors (126, 203, 204). Cer has been shown to trigger photoreceptor degeneration in vitro by activating PARP-1, thus increasing the levels of poly-ADP ribose polymers and promoting translocation of apoptosis-inducing factor (AIF) from mitochondria to the nuclei of photoreceptors, which are established features of Parthanatos. Inhibition of PARP-1 activation protects these neurons from Cer-induced death (55). Interestingly, activation of PARP-1 has been shown in retinas of *rd1* and *rd2* mice, which are animal models of RP. PARP-1 inhibition increases photoreceptor survival in these retinas, supporting Parthanatos as a common death pathway in RP animal models (299, 300, 301). Hence, Cer may be a common arbiter of photoreceptor death in RP through the activation of Parthanatos. Taken together, these studies indicate not only that Cer accumulation triggers photoreceptor death in RP models, but also that inhibiting Cer production protects the retina from degeneration, underscoring the potential of targeting sphingolipid metabolic pathways for developing new therapies for RP. Inhibitors of de novo Cer synthesis, such as FTY720 and myriocin, or of aSMase, such as desipramine, may provide promising therapeutic options for treating diseases that involve Cer accumulation, like RP. FTY720 and desipramine are Food and Drug Administration-approved drugs that have already been tested for their safety; likewise, a novel ophthalmic formulation of myriocin has been shown to be well tolerated and to effectively modulate Cer synthesis (289). Further work is essential to fully unravel the role of Cer in RP and develop efficient tools to modulate its levels for treating this disease.

Findings linking mutation in the CerK-like (CERKL) gene with autosomal recessive RP (302, 303, 304, 305) brought great expectations to the field by suggesting an association between dysregulation of sphingolipid metabolism and RP. Although CERKL is structurally very similar to CerK, it remains as an orphan lipid kinase because its endogenous substrate is still unknown (306, 307). Mandal et al. (152) have shown that CERKL is highly expressed in mature retinas; this expression increases in light-stressed retinas and decreases in retinas of NeuroD1 knockout mice, which develop a rapid postnatal rod and cone degeneration. This suggests that CERKL may be important for the stress response and protection of photoreceptors. Knockdown of CERKL in zebrafish renders retinal cells more sensitive to oxidative stress, whereas its overexpression protects them from apoptosis (308); CERKL regulates the thioredoxin 2 (TRX2) antioxidant pathway maintaining mitochondrial TRX2 in the reduced redox state, and pathological mutations in CERKL that disrupt this interaction may lead to retinal cell death. A recent work demonstrated that CERKL is critical in the regulation of autophagy in the zebrafish retina by stabilizing SIRT1, an NAD-dependent deacetylase; knockdown of CERKL decreased autophagy in both photoreceptors and RPE cells, reducing SITR1 levels (309). Hence, although its connection to sphingolipid metabolism has not been established, CERKL emerges as a critical regulator of different protective pathways in the retina.

### Sphingolipids: unlocking the pathways to fibrotic disorders in the retina?

Fibrosis is a common feature in virtually every organ subjected to a lesion that impairs its architecture, and both acute and chronic injuries from the most diverse etiologies can converge in this process. It is characterized by an excessive production and deposition of extracellular matrix components as well as an increase in the content of mesenchymal cells that end up replacing functional organ cells and tissue. The augmented proportion of mesenchymal versus parenchymal cells is due to exacerbated mesenchymal proliferation, migration, and dedifferentiation, all of which lead to the formation of atypical scars that threaten the normal function of a given tissue. Many cytokines and trophic factors have been reported to promote fibrosis, including tumor growth factor β (TGF-β), platelet-derived growth factor, connective tissue growth factor, IL-3, and S1P (310). S1P and its signaling pathways have recently become a hot spot in the search for new mechanisms involved in the progression of fibrosis (311). A role for S1P in the development of fibrosis has been extensively documented in the liver, with an upregulation of SphK1 activity (312, 313, 314), as well as the kidney (315, 316), heart (317, 318), and lung (319, 320). Opposing data exist regarding the role of Cer and C1P in fibrosis. Although Cer has been shown to attenuate lipopolysaccharide response in macrophages (321), it has also been linked to chronic inflammation in adipose tissue (322), whereas inhibition of aSMase reduces pulmonary fibrosis in cystic fibrosis (323, 324). In turn, C1P displays anti-inflammatory functions in chronic obstructive pulmonary disease, where it attenuates inflammation and fibrosis (325). However, C1P increases blood-brain barrier permeability (326) and has pro-inflammatory attributes in macrophages (327).

In the retina, several diseases such as AMD, DR, and proliferative vitreoretinopathy share an underlying phenomenon of abnormal scar formation. This distorts the structured organization of the retina, impairing visual functionality. Interestingly, the main cells that support retinal homeostasis, Müller glial cells and RPE cells, are also responsible for contributing to the development of fibrotic scars. The involvement of sphingolipids in retinal fibrosis is poorly understood, though there is some evidence implicating S1P and Cer in the process. An approach using anti-S1P antibodies reduced sub-retinal fibrosis in a mouse model of choroidal neovascularization (328) and prevented excessive scar formation in animal models of glaucoma (329). S1P promotes the migration of Müller glial cells, which is exacerbated in fibrotic processes, through S1P synthesis and release followed by the activation of S1PR3 and the later stimulation of ERK/MAPK and PI3K signaling pathways (129). S1P also stimulates myofibroblast transformation, proliferation, and production of pro-fibrotic proteins in human RPE cells (130). These findings demonstrate that S1P promotes processes associated with fibrosis development, supporting the role of S1P as a pro-fibrotic molecule in the retina. S1P may promote retinal fibrosis by enhancing cellular proliferation, migration, and trans-differentiation into myofibroblasts, all of which lead to retinal scar formation. Pursuing these studies will allow us to establish whether S1P is a mediator of these processes.

The connection between Cer and retinal fibrosis is still unclear. Cer accumulation due to excessive aSMase activation has been reported in late stages of AMD (201), and nonpolarized RPE cells, which are frequently observed in proliferative retinopathies, are more susceptible to Cer-induced apoptosis (153). Moreover, photoreceptor apoptosis during experimental retinal detachment, a condition that frequently leads to fibrotic scars, is associated with an increase of Cer production (330). Cer might promote retinal fibrosis in an indirect fashion. As stated earlier, increased Cer triggers photoreceptor death upon different injuries to the retina (202, 203, 204, 278, 285, 286). The consequent massive photoreceptor degeneration may in turn provoke an exacerbated response in RPE and Müller glial cells. In an attempt to repair this damage, these cells may increase their proliferation, migration, and/or trans-differentiation, which would ultimately culminate in the development of a fibrotic scar.

Even though the roles of sphingolipids in retinal fibrosis are barely starting to be explored, they are already emerging as attractive targets to prevent this complication. Sphingolipids such as S1P and C1P participate in many cellular processes such as proliferation, migration, and differentiation, which are also responsible for developing fibrotic scars in the retina. However, they also regulate vital retinal functions including neurotransmitter release, survival, and differentiation of retinal neurons. Additional studies are required to develop sufficient understanding of sphingolipids to utilize them as pharmacological tools in the retina, taking advantage of their beneficial roles while avoiding their deleterious effects.

### Best disease: a role for Cer?

Best disease, also known as vitelliform macular dystrophy (VMD), is a rare (1:15,000) inherited retinal degenerative disease. It affects the macula in both eyes, although not always equally; sharper vision is sometimes retained in one eye. In rare cases, individuals may not experience symptoms. Usually diagnosed during teenage years, vision generally deteriorates later in life. VMD is characterized by accumulation of the yellow pigment, lipofuscin, in the RPE at the center of the retina, which eventually damages the cone cells in the macula, leading to a blurring or distortion of central vision and gradual loss of central visual field (331). Mutations in the human bestrophin-1 (*BEST1*) gene, previously known as the *VMD2* gene, are thought to cause this disease. The protein, human Best1 (hBest1), encoded by this gene, is an integral membrane protein found primarily in the RPE, which functions as an anion channel (332, 333, 334). Mutations in the *BEST1* gene have been uncovered as the cause of several other ocular diseases, including adult-onset macular dystrophy and bull’s eye maculopathy (335). In addition to a nonfunctional hBest1 protein, disruption of processes that regulate hBest1 function can also lead to retinopathies. A defective Ca^2+^-activated Cl^−^ channel in the RPE basolateral membrane, where hBest1 is expressed, can lead to VMD. A PKC phosphorylation site (serine 358) in hBest1 is important for the sustained function of this Cl^−^ channel (334, 336, 337).

Scarce information exists regarding the mechanisms of this disease. Cer buildup in cultured cells due to exogenous addition of Cer or of bacterial SMase leads to the rapid dephosphorylation of serine 358 in hBest1. Exposure to hypertonic stress that activates nSMase has a similar effect, which is prevented by a nSMase inhibitor (manumycin A). This suggests that accumulation of Cer at early stages of the disease may impair hBest1 function, leading to abnormal fluid transport and retinal inflammation (333, 334, 338), and therefore implies that Cer may contribute to the onset of this disease.

### Macular telangiectasia: are deoxysphingolipids involved?

Macular telangiectasia (Mac Tel) type 2 is a rare macular disease with a prevalence of 0.0045–0.06%. The onset of symptoms occurs in the late decades of life and lead to central vision loss (339, 340, 341, 342). This disease has a strong genetic component, as evidenced by extended families having multiple affected members (343). Recent insights from genome-wide association and metabolomic studies suggest that Mac Tel is associated with low serine levels in the blood (344). Serine is a substrate in numerous metabolic pathways, including protein, nucleotide, and lipid synthesis. SPT, which condenses serine and palmitoyl-CoA, is the rate-limiting enzyme in de novo biosynthesis of sphingolipids (345). Mutations in SPT encoding genes *SPTLC1* and *SPTLC2* have been associated with increased synthesis of atypical deoxysphingolipids, which are toxic to multiple cell types, particularly neurons (346, 347, 348). These deoxysphingolipids can also accumulate when levels of serine are low, even in the absence of mutations in *SPTLC1* or *SPTLC2* (349). Most patients with Mac Tel have low serine levels and elevated deoxysphingolipid levels even when they show no variants in *SPTLC1* or *SPTLC2*, suggesting that high levels of atypical deoxysphingolipids may be risk factors for Mac Tel (350). Interestingly, reducing circulating serine increases deoxysphingolipids, which are toxic to human photoreceptors and cause functional defects in mouse retinas; deoxydihydroceramide has been identified as the main neurotoxic species and accounted for nearly 90% of the hydrolyzed deoxysphinganine levels measured in Mac Tel patients (350). Although the low prevalence of Mac Tel accounts for the scarce clinical data on this disease, the existing data support a role for elevated deoxysphingolipid levels in the development of macular disease in Mac Tel patients, as well as other macular dystrophies. Further work is needed to uncover the potential involvement of other sphingolipid metabolites and the mechanisms of retinal toxicity observed in Mac Tel.

### DR: sphingolipids as key triggers of pathogenesis?

DR is a microvascular disease that is one of the most common complications of both type 1 and type 2 diabetes mellitus. DR involves chronic low-grade inflammation resulting in retinal vascular degeneration and defective repair of retinal endothelial cells (351). It is the principal cause of blindness in people between the ages of 20 and 65 (352) and can be expected to develop within 20 years of diabetes mellitus diagnosis (353). Nonproliferative DR is the early form and is characterized by various microvascular abnormalities including vessel occlusion and microaneurysms. Although nonproliferative DR can exist asymptomatically for years, it can cause vision loss through macular edema and is capable of rapid progression to proliferative DR (PDR), the more debilitating form of the disease (354). PDR is characterized by proliferation of blood vessels into the retina, eventually leading to vision loss (293). The pro-inflammatory cytokines and VEGF secreted by RPE and activated retinal glial cells have been reported to contribute to damage of retinal vasculature (351, 355, 356). In addition, inflammation is further exacerbated by increase in leukocyte adhesion by activation of circulating myelomonocytic cells from bone marrow and myeloid-derived monocyte infiltration (357, 358). This in turn activates resident microglia, astrocytes, and Müller glia in the retina, leading to chronic inflammation (351, 359, 360). Furthermore, leukostasis of the retinal vasculature has been suggested to be an important contributor of ischemia and endothelial damage leading to ocular inflammation (361).

Over the years, multiple studies have associated increased levels of sphingolipids, particularly the bioactive sphingolipid Cer, with various aspects of DR. In human and animal models, secreted pro-inflammatory cytokines cause retinal endothelial cells to secrete aSMase, which increases Cer levels due to hydrolysis of SM (362). It has also been shown that increase in aSMase by TNF-α and IL-1β induce VEGF and ICAM-1 in human retinal endothelial cells and regulate retinal microangiopathy (363). In addition, sphingolipid composition of type 2 diabetic and nondiabetic postmortem human retinas show increases in total Cer, LacCer, and SM in diabetic vitreous samples (364). These studies underscore the likely involvement of sphingolipids in DR-associated pathologies of the retina, retinal vessels, vitreous, and surrounding tissues. In DR pathogenesis, apoptosis of pericytes is an early event, resulting from the increased concentration of the saturated free fatty acid, palmitate, a consequence of sustained hyperglycemia. Incubation with palmitate increases Cer levels in cultured pericytes, leading to apoptosis and inhibition of CerS. Overexpression of ASAH1 reverses the proapoptotic effects of palmitate, suggesting a role of Cer in the early pathogenesis of DR (365). Animal models of streptozotocin-induced diabetes show a decrease in Cer levels with a concomitant increase in GlcCer. As abnormal GlcCer accumulation can cause mitochondrial, endoplasmic reticular, and endo-lysosomal dysfunction, GlcCer might be an important player in DR pathology and retinal cell death (240, 241). In vitro studies have shown that GlcCer increases in retinal neurodegeneration and hyperglycemic retinal neurons, while inhibition of GCS increases the viability and insulin sensitivity of retinal neuronal cells (166, 176, 362, 366). In addition, in Zucker diabetic fatty rats, pharmacological inhibition of GCS has been shown to increase insulin sensitivity (367). LacCer may also play a role in DR, as suggested by its role in inflammation (368) and VEGF-mediated angiogenesis (369). Retinal vascular permeability is mediated by very long-chain Cers, and their increase stabilizes tight junctions and prevents blood-retinal barrier dysregulation in in vitro models. Elongation of very long-chain fatty acids protein 4 (ELOVL4), which is responsible for the synthesis of the very long-chain fatty acids incorporated in very long-chain Cer (370, 371), is highly reduced in streptozotocin -induced diabetic rats (370), and overexpression of retinal ELOVL4 has been shown to decrease endothelial permeability in a bovine retinal endothelial cell model (371). S1PR2 has been implicated in the extensive neovascularization that occurs in PDR. An in vivo model of ischemia-induced retinopathy showed that neovascularization is significantly reduced in S1PR2-deficient mice (212). Choroidal and subretinal neovascularization in mouse models is also significantly inhibited by blocking S1P with the therapeutic antibody, sonepcizumab (213).

Collectively, these studies show substantial sphingolipid involvement, mostly of Cer, GlcCer, LacCer, and S1P, in the pathogenesis of DR. They also underscore the importance of understanding and potentially exploiting sphingolipid bioactivity in DR for the development of a new generation of therapeutic agents.

## Summary and future directions

Although still enigmatic, sphingolipids have by now established themselves as leading actors in the pathophysiology of retinal diseases. Cer consistently acts as a key signal to activate neuronal death in multiple retinal degenerative diseases, including AMD, glaucoma, RP, and DR. S1P has more ambivalent functions, preventing neuronal death but also promoting inflammation, cell migration, fibrosis, and neovascularization in AMD, glaucoma, and pro-fibrotic disorders. Cer, S1P, C1P, and GM1 may be critical players in the onset and progression of retinal inflammation, a central event in most retinopathies leading to visual loss. Excitingly, their metabolic pathways and modulation of the sphingolipid rheostat are emerging as promising targets for the development of new strategies for the treatment of retinal diseases.

However, there are still crucial knowledge gaps to be addressed before trying to paint an all-encompassing picture of ocular sphingolipid bioactivity. Data regarding the localization of sphingolipid receptors and metabolic enzymes in the retina, for instance, is still sparse. SphK1 and -2 and S1PR1–3 have been immunohistochemically characterized in rat eyes and were found to have distinct areas of localization across different retinal layers and cell types (212, 214). Additionally, CERKL has been shown to localize to the retinal ganglion cells, inner nuclear layer, RPE, and photoreceptor inner segments in rat eyes (152). However, due to paucity of more detailed information in this area, there is still very inadequate understanding of the overall sphingolipid metabolic gradient across the retina. Delineating the patterns and regulation of sphingolipid metabolism through enzyme localization studies in the retina is one of several areas that would benefit from further committed investigation. There is also little information available regarding sphingolipid composition of the various retinal cell types and layers. Segmental compositional analyses of retinal layers/cell types using advanced laser-capture technology and MALDI-TOF-MS would be a valuable contribution to the current knowledge base. Similarly, a thorough evaluation of the alterations in sphingolipid composition and metabolism in retinas affected by diverse pathologies would provide the knowledge to comprehend how these changes contribute to pathological scenarios.

As stated above, currently available information positions Cer as a critical arbitrator of neuronal death in retinopathies and highlights the relevance of pharmacological manipulation to prevent its accumulation and preserve neuronal functions. Pursuing these studies in vivo would corroborate the usefulness of this therapeutic approach and contribute to identifying novel therapeutic tools. Existing knowledge on S1P functions in the retina sheds light not only on its potential involvement in neuronal survival but also in inflammation, vascular development, migration, and dedifferentiation, all being key features of devastating retinopathies. Given the established roles played by S1P in diverse diseases, resolving the puzzle of the role of S1P in retinal physiology and pathophysiology is an imperative concern in the field. Additionally, the part played by C1P as an inflammatory mediator in several retinopathies remains to be established.

Future work will contribute to unraveling the complex network of sphingolipid metabolism and provide additional mechanistic insights regarding how various sphingolipids participate in the progression of retinal pathologies, thus bringing about new tools for future therapeutic development for these diseases.

## Conflict of interest

The authors declare that they have no conflicts of interest with the contents of this article.
